# Exogenous 24-epibrassinolide promoted growth and nitrogen absorption and assimilation efficiency of apple seedlings under salt stress

**DOI:** 10.3389/fpls.2023.1178085

**Published:** 2023-04-14

**Authors:** Bo Yu, Laiping Wang, Qiuzhu Guan, Xiaomin Xue, Wensheng Gao, Peixian Nie

**Affiliations:** ^1^ Shandong Institute of Pomology, Shandong Key Laboratory of Fruit Biotechnology Breeding, Taian, China; ^2^ College of Horticulture, Shenyang Agricultural University, Shenyang, China; ^3^ Shandong Provincial Department of Agriculture and Rural Affairs, Shandong Agricultural Technology Extension Center, Jinan, China

**Keywords:** M9T337 seedlings, 24-epibrassinolide, NaCl stress, C and N assimilation, NUE

## Abstract

**Introduction:**

High salinity significantly hampers global agricultural productivity. Plants typically undergo lower nitrogen utilization efficiency (NUE) under salt stress. As an active byproduct from brassinolide biosynthesis, 24-epibrassinolide (EBR) is involved in regulating the stress-treated plant N absorption and assimilation. However, the exogenous EBR application effects’ on N absorption and assimilation in apple exposed to the salt-stressed condition remains unclear.

**Methods:**

We sprayed exogenous EBR (0.2 mg L^−1^) on apple dwarf rootstock (M9T337) seedlings (growing hydroponically) under salt (NaCl) stress in a growth chamber. We analyzed the seedling development, photosynthesis and its-mediated C fixation, N ( ${\text{NO}}_{3}^{-}$ ) absorption and assimilation in reponse to exogenous EBR application under salt stress.

**Results:**

The findings demonstrated that NaCl stress greatly hampered seedlings’ root growth and that exogenous EBR application obviously alleviated this growth suppression. Exogenous EBR-treated plants under NaCl stress displayed the more ideal root morphology and root activity, stronger salt stress tolerance and photosynthetic capacity as well as higher C- and N-assimilation enzyme activities, ${\text{NO}}_{3}^{-}$ ion flow rate and nitrate transporter gene expression level than did untreated plants. Furthermore, the results of isotope labeling noted that exogenous EBR application also enhanced ^13^C-photoassimilate transport from leaves to roots and ^15^ ${\text{NO}}_{3}^{-}$ transport from roots to leaves under NaCl stress.

**Conclusion:**

Our findings imply that exogenous EBR application, through strengthening photosynthesis, C- and N-assimilation enzyme activities, nitrate absorption and transport as well as synchronized optimizing the distribution of seedlings’ C and N, has a fundamental role in improving NUE in apple rootstock seedlings under salt stress.

## 1 Introduction

Soil salinity is a major environmental stressor for agricultural production, and soil salt stress is becoming increasingly severe due to global climatic changes and unsustainable agricultural practices (e.g., improper irrigation) (Shabala, 2009; Nazar et al., 2011; Krishnamurthy et al., 2016; Li et al., 2017; Betzen et al., 2019). The Loess Plateau is one of the largest and most ideal apple production regions in China. However, a large percentage of the orchard land of the Loess Plateau is being suffered from soil salt stress, which can severely restrict the growth and nutrient utilization efficiency of apple and then causing enormous losses for orchardists (Kawanabe and Zhu, 1991; Jia et al., 2019; Su et al., 2020). As the one of crucial macronutrient in apple production, appropriate N supplies considerably aids in apples’growth and development and elevate its economic value (Xu et al., 2020). However, due to the single-minded quest for high yields and large-sized fruits, over usage of N fertilizer has become prevalent in the apple orchards on the Loess Plateau, and the input of N fertilizer is considerably greater than the requirement of the trees (Zhu et al., 2022). Furthermore, apple trees is not able to efficiently utilize the applied N fertilizer. The excessive application of N fertilizer and low N utilization efficiency (NUE) can increase the production cost for fruit growers and indirectly contribute to a range of ecological and environmental problems (Chen et al., 2017; Chen et al., 2018; Ge et al., 2018; Wang et al., 2020a), and this problem is exacerbated by soil salt stress due to its inhibitory action on N absorption and assimilation (e.g., by having a detrimental effect on root development or weakening leaf photosynthetic capacity) (Yin et al., 2010; Khan et al., 2011; Jia et al., 2020). Therefore, it is a main issue for producers on the Loess Plateau to explore the methods of boosting the N uptake of the salt-stressed apple.

As an active by-product from brassinolide biosynthesis, 24-epibrassinolide (EBR) has been proved to have the capacity to drive a variety of metabolic processes in plants, such as generation of nucleic acids, protein production, and photosynthesis (Sairam, 1994; Bajguz, 2000; Kanwar et al., 2017; Tanveer et al., 2018; Peres et al., 2019; Ahammed et al., 2020). Along with playing a positive function in typical plant development and growth, EBR exhibits anti-stress effects that help to reduce the detrimental effects of various abiotic stresses on plants, especially in the enhancement of plant tolerance to salt stress toxicity (Krishna, 2003; Talaat and Shawky, 2012; Yusuf et al., 2012; Abbas et al., 2013; Duran et al., 2017; Soylemez et al., 2017). For instance, treatment with EBR was demonstrated to alleviate the growth reduction of *Fragaria ananassa* exposed to the salt stress by reducing the ion injury (Karlidag et al., 2011), enhance the osmolyte accumulation of *Pisum sativum* grown in a high salt environment (Shahid et al., 2014) as well as augment the tolerance of *Triticum aestivum* to salt stress damage by regulating the antioxidant defense systems (Shahbaz and Ashraf, 2008). Furthermore, researches on *Solanum melongena* (Wu et al., 2012) and *Vigna radiata* (Mir et al., 2015) showed that EBR could effectively improve plants’ salt tolerance through strengthening its photosynthetic capacity.

The macronutrient N is indispensable for the plants’ optimal development and growth, enhancing the absorption and assimilation of N is essential for improving the NUE of plants (Bai et al., 2016; Zhao et al., 2016; Chen et al., 2018; Xing et al., 2021; Zhu et al., 2022). However, the N absorption and assimilation of plant could be easily affected by various stress environment conditions (Abdelgadir et al., 2005; Shu et al., 2016; Liang W et al., 2018). Existing studies have documented the regulatory mechanisms of exogenous EBR treatment on plants’ N absorption and assimilation under stress conditions, such as alleviating the reduction of ${\text{NO}}_{3}^{-}$ flux in cucumber roots induced by sub-optimal root zone temperatures (Anwar et al., 2019), enhancing the N assimilation-related enzyme activities of the salt stress treated chickpea (Wani et al., 2017), and regulating NRT genes expression level (*ZmNRT2.1*, *ZmNRT2.2*) in maize exposed to the low ${\text{NO}}_{3}^{-}$ environment (Xing et al., 2022). Contrarily, very few studies have focused the effects of exogenous EBR on the N absorption and assimilation of apple under stress condition. Owing to its exceptional health benefits and economic value, apple has been extensively cultivated worldwide. China has the highest apple cultivation area and yields globally (Yang et al., 2021). Although exogenous EBR application could increase apple seedlings’ N content grown in the salt stress environment (Su et al., 2020; Zheng et al., 2022), specifics regarding exogenous EBR’s effects on the uptake and assimilation of N in apple treated by salt stress are still unclear, particularly from the perspective of the coordinated control of C and N assimilation. Previous studies have proved that the enhancement of C and N assimilation is of vital importance for the normal operation of physiological and biochemical processes in plants (Reguera et al., 2013; Ren et al., 2021), and the plants’ N uptake and assimilation efficiency were strongly associated with changes in photosynthesis, photosynthesis-mediated C fixation and the transport of photosynthate (Hu et al., 2017; Erdal, 2019; Ren et al., 2020).

Based on the findings of existing research, this study was actualized in a growth chamber at Shenyang Agricultural University, we sprayed exogenous 2,4-epibrassinolide (0.2 mg L^−1^ EBR) on apple dwarf rootstock (M9T337) seedlings (an extensively applied apple rootstock) to focus on the effects of exogenous EBR on N absorption and assimilation by M9T337 seedlings under salt stress conditions (NaCl stress). Nitrate was selected as the only N source in this trial. We hypothesized the positive regulation of EBR with regard to alleviating the inhibition induced by salt stress on seedlings’ C and N assimilation, and then improving the NUE in seedlings. The results observed in this study may shed new light on the improvement of NUE in salt-stressed apple orchards.

## 2 Materials and trial methods

### 2.1 Plant materials and applied treatments

In 2021, seedlings of M9T337, a widely applied apple dwarf rootstock-were raised in a plant growth chamber under conditions that mimicked those of nature (natural lighting, temperature settings of 22-27°C for day and 4-9°C for night, and relative humidity of 50-60%). When the rootstocks reached a height of about 15 cm, those displaying similar growth were chosen and transplanted into plastic basins (45 cm × 30 cm × 15 cm) with eight holes, and each hole received a single seedling. For a week, each basin received six liters of half-strength Hoagland’s (Hoagland and Arnon, 1950) solution in order to progressively acclimate to the nutrient solution before transferring to the full-strength solution. The solution was renewed every three days, and to provide support to the seedlings, all the basins were enclosed with a foam board.

Twelve days after transplantation, the treatments were conducted on the seedlings. The concentration of EBR applied in this study was set as 0.2 mg L^−1^ according to our preliminary study (unpublished). Three different treatments were applied to the seedlings. In treatment 1 (control), the seedlings received only full-strength Hoagland’s solution; in treatment 2 (SS treatment), the seedlings were exposed to a salt stress condition *via* receiving full-strength Hoagland’s solution along with 100 mM NaCl; as for treatment 3 (SS+EBR treatment), the seedlings received the same salt stress condition as in treatment 2 in addition to being sprayed with 0.2 mg L^−1^ of EBR (RealTimes, CAS: 7882143-19). Every three days, the nutrient solution was changed for all the treatments, and exogenous EBR was sprayed. Each treatment was divided into two group of three replicates, the one group was for ^15^N and ^13^C labeling (labeling group), another was for other indexes (normal group). The concentrations of nutrient elements in the full-strength Hoagland’s solution remained consistent among all treatments, and the details were as follows: 6 mM K_2_SO_4_, 5 mM Ca(NO_3_)_2_, 2 mM MgSO_4_, 1 mM NaH_2_PO_4_, 0.1 mM EDTA-Fe, 37 µM H_3_BO_4_, 9 µM MnCl_2_·4H_2_O, 0.76 µM ZnSO_4_·7H_2_O, and 0.3 µM CuSO_4_·5H_2_O. The EBR sprayed in this experiment was made into a stock liquor by dissolving it in ethanol before being adjusted to 0.2 mg L^−1^ ultimate concentration. In order to maintain uniformity and ensure that the observed differences were a result of treatment effects, the same amount of ethanol was applied to the other two treatments. The plant materials were harvested after a total of 15 days of treatment (during which the exogenous EBR was sprayed five times and the nutrient solution was renewed five times).

### 2.2 Measurement of dry matter weight

The seedlings were collected after being treated for 15 days and separated into three parts, including roots, stems, and leaves. The measurement of dry matter weight was followed the methods reported by Sha et al. (2019). In brief, following a series of rinsing steps using tap water, detergent, deionized water, and 1% HCl, the plant materials were initially dried for 0.5 hour at 105°C and then left to dry for 72 hours at 80°C. A 1/1,000 electronic balance was used to measure the dry weight of all plant parts.

### 2.3 Analysis of root morphology and root activity

In order to examine the root total length and total surface area, three rootstocks were chosen from each treatment randomly and rinsed using deionized water to remove any contaminants. The root samples were examined with the aid of the WinRHIZO software (Regent Instruments Canada, Inc.). Upon harvest of the seedlings, their root activity in each treatment was estimated by the triphenyltetrazolium chloride (TTC) reduction technique (Chen et al., 2018).

### 2.4 Quantification of the levels of H_2_O_2_, ${\text{O}}_{2}^{•–}$ , malondialdehyde and proline as well as the determination of Na^+^: K^+^

The methods reported by He et al. (2020) were employed to conduct the measurement of hydrogen peroxide (H_2_O_2_), superoxide ions ( ${\text{O}}_{2}^{\cdot -}$ ) and malondialdehyde (MDA) contents as well as the contents of proline in leaves. The quantification of leaves’ Na^+^: K^+^ was followed the methods described by Zheng et al. (2021).

### 2.5 Determination of gas exchange variables, chlorophyll fluorescence parameters, and total chlorophyll

After 15 days treatment period, the fourth leaf from the plant’s apex on the main stem was chosen for measuring the *P*_n_ and *G*_s_ (from 9:00 until 11:30 AM) using the LI-6400XT portable photosynthesis system (LI-COR, Lincoln, NE, United States). Three measurements were taken for each leaf. During the same time frame, the same leaf’s chlorophyll fluorescence parameters were measured with a pulse-modulated chlorophyll fluorescence meter (PAM 2500, Walz, Germany), and three measurements were taken for each leaf. Moreover, the method outlined by Hussain et al. (2019) was employed in this study to the calculation of the value of total chlorophyll content.

### 2.6 Measurement of antioxidant enzyme (SOD, POD and CAT), Rubisco, NR, GS, and GOGAT activities

The enzyme activities of superoxide dismutase (SOD), peroxidase (POD) and catalase (CAT) were determined according to the method of He et al. (2020). Rubisco (Ribulose-1,5-biphosphate carboxylase-oxygenase) was measured, and the measurement were referred to the methods obtained by Hu et al. (2016a), respectively. The nitrate reductase (NR), glutamine synthetase (GS), and glutamate synthase (GOGAT) activities were estimated based on methods outlined by Hu et al. (2016b).

### 2.7 Determination of roots’ NO_3_^−^ flow rate

The roots’ NO_3_^−^ flow rate in this study was analyzed and was measured by a non-invasive micro-test system (NMT 100 Series, USA). In brief, the entire roots of the seedlings were cleaned adequately using deionized water, and about 2 cm of roots (from the root tip) were selected and positioned on a strip of filter paper on a plastic plate. The strip was fastened in place using a tiny block of glass. The roots were immersed in a test solution with a pH of 6.0, and the details of the test solution were as follows: 0.625 mM KH_2_PO_4_, 0.5 mM MgSO_4_, 0.25 mM KNO_3_ and 0.25 mM Ca(NO_3_)_2_. The analysis was initiated once the ${\text{NO}}_{3}^{-}$ flow rate on the root surface had stabilized. The measurements revealed that the seedlings from the various treatments had the highest ${\text{NO}}_{3}^{-}$ ion flow velocity in the closely packed region of the root hairs, located roughly 8 mm from the tip of the root. As a result, individual samples were chosen at random for a subsequent measurement, and data was gathered from ten different positions with five samples for each treatment. To collect the data, the measurement process took 10 min (Xu et al., 2020), and the data were examined with MageFlux (imFluxes v 2.0). Positive readings indicated ${\text{NO}}_{3}^{-}$ outflow (efflux), whereas negative readings indicated ${\text{NO}}_{3}^{-}$ inflow (influx).

### 2.8 ^15^N and ^13^C labeling method and isotope analysis

During the 15 days treatment period, seedlings of label group from each treatment were chosen and grouped together for ^15^N labeling. Whenever the nutrient solution was renewed, 0.5 g of Ca(^15^NO_3_)_2_ (abundance of 10.14%) was introduced to the nutrient solution for ^15^N labeling (the total dosage received by the eight seedlings during five additions was 2.5 g). The rootstocks were harvested and then divided into roots, stems, and leaves after being treated for 15 days for ^15^N analysis. After collection, the samples were placed inside paper bags and dehydrated for 72 hours at 80°C. The abundances of ^15^N and ^13^C were then quantified using MAT-251-Stable Isotope Ratio Mass Spectrometer after pulverizing and filtering the samples through a mesh screen (0.25 mm).

After 12 days of treatment, the seedlings that were used for ^15^N labeling were additionally labeled with ^13^C. The seedlings from each treatment were placed in a sealed marking room (temperature was maintained between 27 to 33°C) together with markers (Ba^13^CO_3_, manufactured by Shanghai Institute of Chemical Technology; abundance of 98%; the dosage received by each seedling was 0.2 g) and fans. Work on marking began at 9:00 AM and continued for four hours. The CO_2_ concentration was kept constant by injecting 5 mL of hydrochloric acid (1 mM) every 30 min with a syringe. Three additional seedlings (normal group) were utilized as a blank control (natural abundance of ^13^C). 72 hours after ^13^C labeling, the samples were harvested for ^13^C quantification analysis. The ^15^N and ^13^C were calculated according to the formulas as follows.

Estimation of ^15^N (according to Xu et al. (2022))


(1) $$\text{Ndff(\%)=}\frac{\text{abundance of}{\;}^{15}\text{N in plant -natural abundance of}{\;}^{15}\text{N}}{\text{abundance of}{\;}^{15}\text{N in fertilizer -natural abundance of}{\;}^{15}\text{N}}\times 100\%$$

(2) $${\;}^{15}\text{N absorbed by each organ=Ndff(\%)}\times \text{total N content(mg)}$$

(3) $${\;}^{15}\text{N partitioning rate(\%)=}\frac{{\;}^{15}\text{N absorbed by each organ from fertilizer (g)}}{\text{total}{\;}^{15}\text{N absorbed by plant from fertilizer (g)}}\times 100\%$$

Calculation of ^13^C (according to Wang et al., 2020b)


(4) $$\text{Abundance of}{\;}^{13}\text{C: }{\text{F}}_{i}\text{(\%)= }\frac{{(\delta }^{13}{\text{C}+1000)\times \text{R}}_{\text{PBD}}}{{(\delta }^{13}{\text{C}+1000)\times \text{R}}_{\text{PBD}}+1000}\times 100\%$$

R_PBD_ in formula (4) means the standard ratio of carbon isotope, and the value of R_PBD_ is 0.0112372


(5) $${\text{Carbon content of each organ: C}}_{\text{i}}\text{= organ dry matter }\left(\text{g}\right)\times \text{ organ total carbon content }\left(\%\right)$$

(6) $$\text{Content of}{\;}^{13}\text{C in each organ:}{\;}^{13}{\text{C}}_{i}\text{(mg)=}\frac{{\text{C}}_{i}{\times \text{(F}}_{i}{\text{-F}}_{nl})}{100}\times 1000$$

F*_nl_* in formula (6) means the natural abundance of ^13^C of each organ


(7) $${\;}^{13}\text{C partitioning rate:}{\;}^{13}\text{C(\%)=}\frac{{\;}^{13}{\text{C}}_{i}}{{\;}^{13}{\text{C}}_{\text{net absorption}}}\times 100\%$$

### 2.9 Extraction of RNA and analysis of gene expression

The transcript levels of genes’ were investigated. In brief, the total RNA of sample was extracted and purified according to the operation guide of plant RNA extraction kit (R6827, Omega Bio-Tek, Norcross, GA, USA), and then a NanoDrop 2000 spectrophotometer (Thermo Fisher Scientific, Waltham, MA, USA) and agarose gel electrophoresis were employed to detect samples’ total RNA concentration and its purity, respectively. Subsequently, 1 μg total RNA was utilized to produce first-strand cDNA using a PrimeScript RT Reagent Kit with gDNA Eraser (DRR037A, Takara, Dalian, China) in a final volume of 20 μl. Quantitative PCR was carried out for each gene with 10 μl of 2× SYBR Green Premix Ex Taq II (DRR820A, Takara), 0.5 μl of cDNA, and gene-specific primer at 0.2 μM. *β-actin* was selected as the reference gene in this study, the details of gene-specific primer and *β-actin* were listed in **Table S1**. The PCR yields’ homogeneity was substantiated *via* a melting curve program. The 2^−△△Ct^ technique was employed to calculate the relative mRNA expression. Moreover, three technical and three biological replicas were conducted in these qRT-PCR experiments.

### 2.10 Statistical analysis

All results are presented as means ± SD. SPSS 17.0 (IBM, United States), a statistics software, was used to analyze the data collected from this study, using one-way factorial analysis of variance (ANOVA). In all cases, differences were deemed significant at a probability level of *P*< 0.05. Origin 8.0 software was used to drew the figures.

## 3 Results

### 3.1 Root morphology and dry matter of seedling

**Figure 1:**
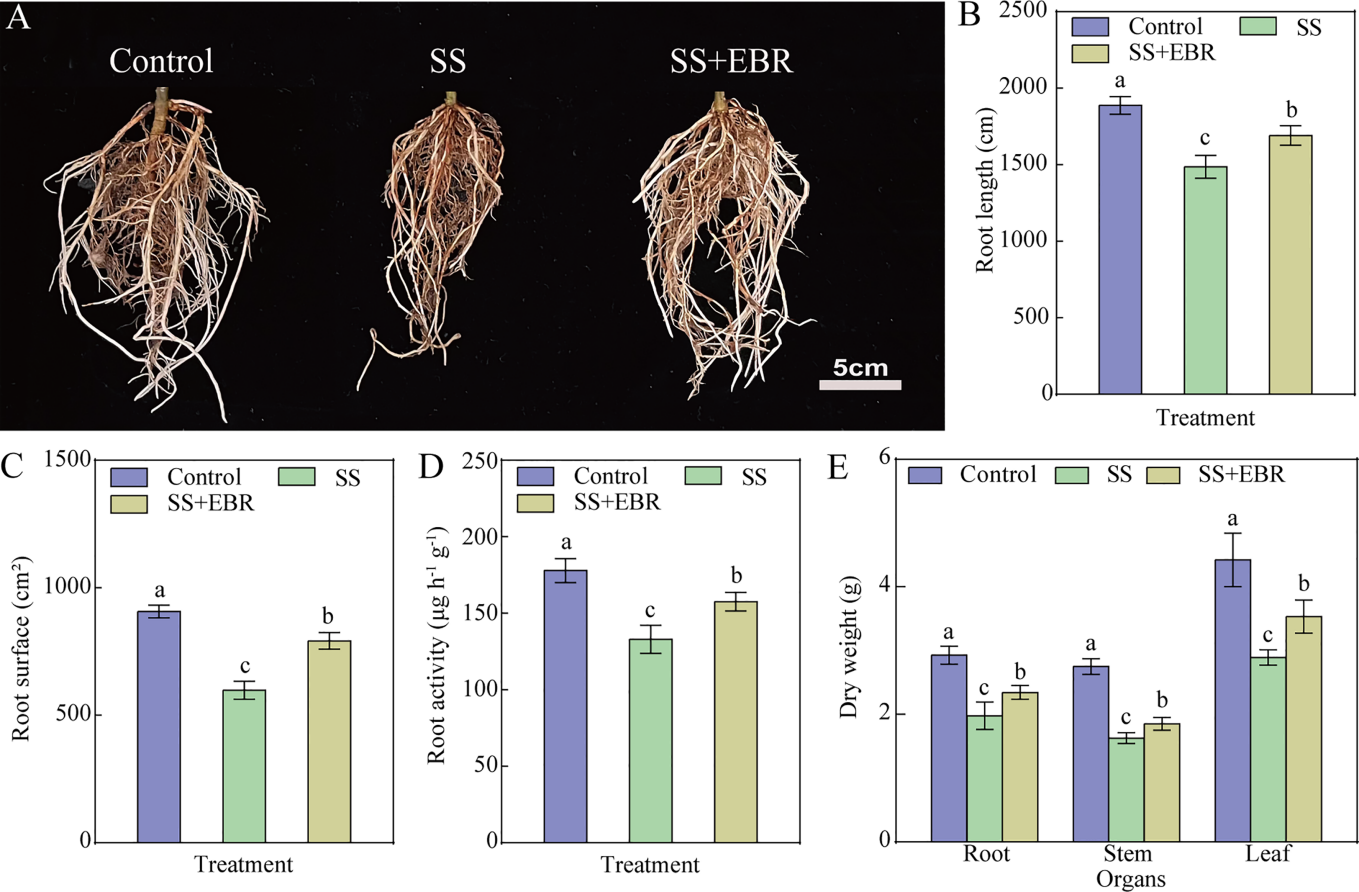
Root phenotype **(A)**, root length **(B)**, root surface **(C)**, root activity **(D)** and dry weight of seedling **(E)** under various treatments. The error bars represent the SD, and the different letters indicate significant differences (*P*< 0.05).

The root morphology of the seedlings differed significantly under diverse treatments (**Figure 1A**). Among all the treatments, the seedlings under SS treatment had the lowest root length and the lowest root surface areas. Exogenous EBR application, however, clearly mitigated the reduction in root total length and root surface area brought on by SS treatment, which were 1.14 times (root length) and 1.32 times (root surface areas) in comparison to those of the SS treatment (**Figures 1B, C**). The root activities showed a similar variation tendency as the roots’ length and surface area (**Figure 1D**).

Additionally, we analyzed the dry matter weight of each plant part. As depicted in **Figure 1E**, regardless of NaCl stress and exogenous EBR application, the highest dry matter weight was observed in leaves, followed by roots and the stems’ was the lowest. Despite the fact that the seedlings exhibited the best growth under control conditions, exogenous EBR application lessened the reduction in each plant part’s dry biomass brought on by salt stress. The dry weight of each part of the rootstock seedlings under the SS+EBR treatment exhibited a significant increase (18.18% (root), 13.50% (stem), 22.15% (leaf)) as compared to that of the SS treatment.

### 3.2 Salt stress tolerance-related index

**Figure 2:**
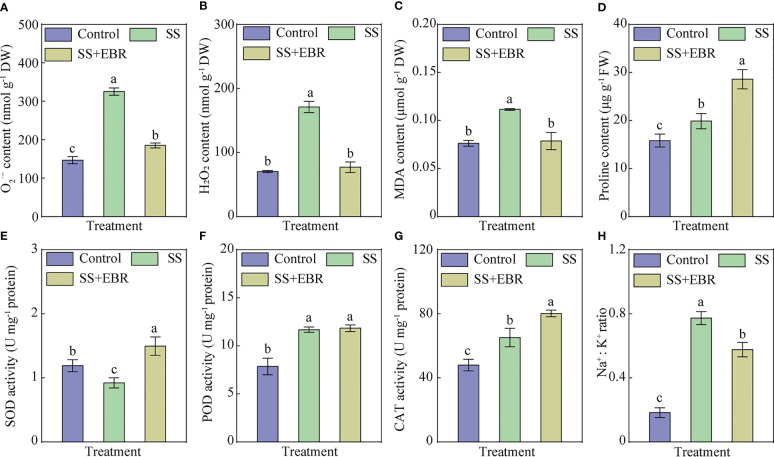
${\text{O}}_{2}^{\cdot -}$ content **(A)**, H_2_O_2_ content **(B)**, MDA content **(C)**, proline content **(D)**, and the activities of SOD **(E)**, POD **(F)** and CAT **(G)** as well as Na^+^: K^+^ ratio **(H)** under various treatments. The error bars represent the SD, and the different letters indicate significant differences (*P*< 0.05).

The contents of H_2_O_2_, ${\text{O}}_{2}^{\cdot -}$ and MDA in leaves were obviously elevated under SS treatment in comparison to those of the control. However, the contents of H_2_O_2_, ${\text{O}}_{2}^{\cdot -}$ and MDA significantly decreased when exogenous EBR was applied under salt stress, compared with SS treatment (**Figures 2A–C**).

The leaves’ SOD, POD, and CAT activities were analyzed. As illustrated in **Figures 2E–G**, compared with control, SS treatment obviously decreased the activity of SOD and elevated the activities of POD and CAT. When exogenous EBR was applied under salt stress, the activities of SOD and CAT were obviously strengthened, compared to SS treatment. However, no significantly difference was observed the change of POD activity between SS and SS+EBR treatment.

We also measured the content of proline under different treatment (**Figure 2D**). The highest proline content was observed in SS+EBR treatment, whilst the lowest was existed in control. Moreover, among all the treatments, the SS treatment resulted in the highest Na^+^: K^+^ ratio. Compared with SS treatment, SS+EBR treatment obviously decreased the value of Na^+^: K^+^ ratio (**Figure 2H**).

### 3.3 Chlorophyll content and photosynthetic-related parameters

**Figure 3:**
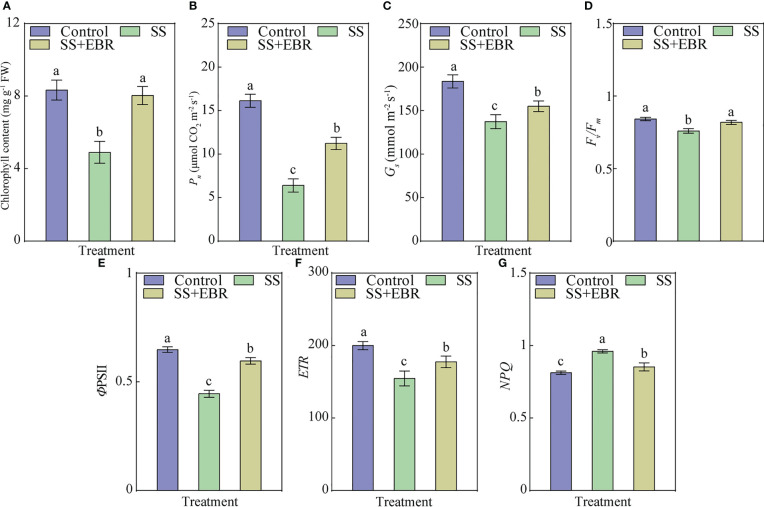
Chlorophyll content **(A)**, *P*_n_ **(B)**, *G*_s_ **(C)**, *F*_v_/*F*_m_ **(D)**,*Φ*PSII **(E)**, *ETR* **(F)** and *NPQ* **(G)** under various treatments. The error bars represent the SD, and the different letters indicate significant differences (*P*< 0.05).

As illustrated in **Figure 3A**, the value of chlorophyll content under SS treatment was only 4.9 mg g^−1^ FW, a drop of 41.18% from the control. The chlorophyll content recovered to the control level when exogenous EBR was administered.

In this experiment, after 15 days of treatment, we monitored the *P*_n_ of leaves receiving various treatments. The SS treatment resulted in the lowest *P*_n_ of leaves, 60.32% lower than the control. However, when exogenous EBR was applied, the value of *P*_n_ was 1.75 times more than in the SS treatment plants (**Figure 3B**). We also founded that the value of *G*_s_ was increased by 12.87%, when EBR treatment was provided under NaCl stress, compared to the SS treatment (**Figure 3C**).

In comparison to the control, the values of *F*_v_/*F*_m_, *Φ*PSII and *ETR* were both obviously decreased by SS treatment. The seedlings under the SS+EBR treatment, however, showed significantly higher *Φ*PSII and *ETR* values than those under the SS treatment, which were 1.33 and 1.15 times in comparison to those of the SS treatment, despite the fact that these values were still lower than those under control (**Figures 3D–F**). Additionally, SS treatment significantly raised the value of *NPQ* in the seedling leaves, whereas exogenous EBR application reduced it (**Figure 3G**).

### 3.4 C and N assimilation-related enzymes activities

**Figure 4:**
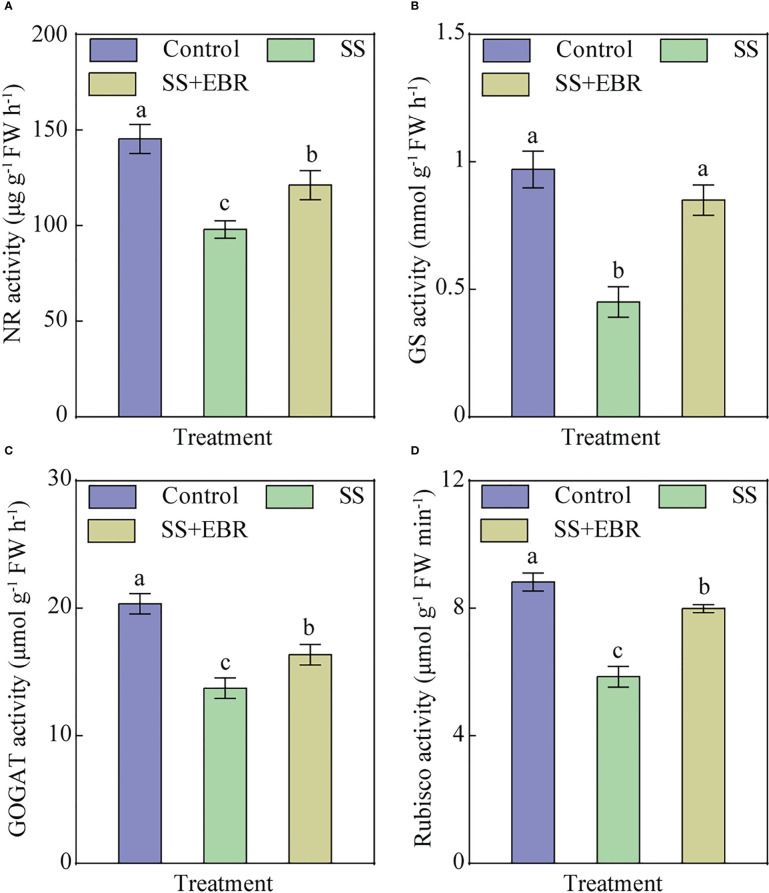
NR **(A)**, GS **(B)**, GOGAT **(C)** and Rubisco **(D)** activities under various treatments. The error bars represent the SD, and the different letters indicate significant differences (*P*< 0.05).

The activities of NR, GS, and GOGAT were greatly reduced in the SS-treated leaves; their levels were only 0.67, 0.46, and 0.67 times those of the control, respectively. However, following a 15-day SS+EBR treatment, there was a significantly smaller decline in these N metabolism-related enzyme activities compared to the SS treatment. The NR, GS, and GOGAT activities rose by 23.67%, 88.90% and 19.23%, respectively, over those in the only SS treatment (**Figures 4A–C**).

We examined the influence of exogenously applied EBR on Rubisco activity. After 15 days of SS treatment, Rubisco activity was markedly decreased, only 0.66 times that of the control. Exogenous EBR application, on the other hand, decreased the inhibition of Rubisco activity brought on by salt stress (**Figure 4D**).

### 3.5 ^13^C accumulation and ^13^C distribution ratio

**Figure 5:**
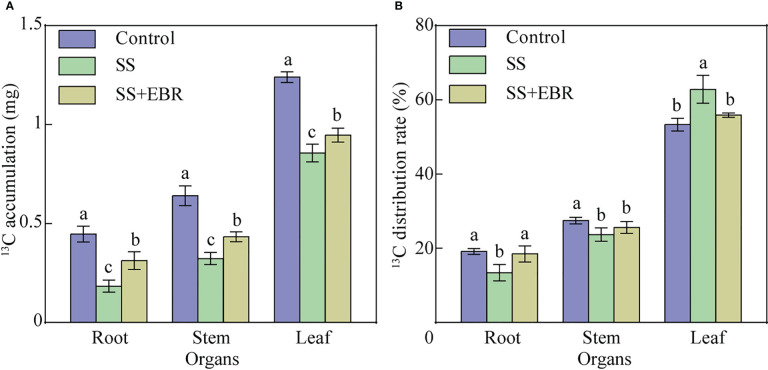
^13^C accumulation **(A)** and ^13^C distribution **(B)** under various treatments. The error bars represent the SD, and the different letters indicate significant differences (*P*< 0.05).

Regardless of the treatment, the highest ^13^C accumulation was detected in the leaves, followed by stems and the lowest was observed in roots. Following three days of ^13^C labeling, the ^13^C accumulation in the seedlings under diverse treatments varied significantly. Among all treatments, the SS treatment resulted in the lowest accumulation of ^13^C in each plant organ of the rootstock seedlings. The accumulation of ^13^C in all organs of the seedlings were greater under the SS+EBR treatment than that under the SS treatment. However, the highest ^13^C accumulation in all organs of the seedlings were still observed in control, which were 1.45 (root), 1.49 (stem) and 1.31 times (leaf) than that under the SS+EBR treatment (**Figure 5A**).

The ^13^C distribution rates in all plant parts of the seedlings followed the same pattern as ^13^C accumulation, in the order of root<stem<leaf. The lowest ^13^C distribution rate of roots was observed in the SS treatment. An opposite trend was, however, observed in the leaves. The SS+EBR treatment obviously elevated ^13^C distribution rate in the roots compared to the SS treatment. Moreover, no significantly differences were observed in the changes of ^13^C distribution rate in the roots and leavers between SS+EBR and control.

### 3.6 ^15^N accumulation and ^15^N distribution ratio

**Figure 6:**
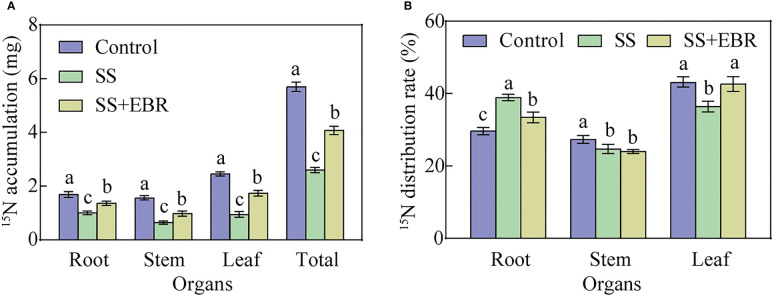
Effects of different treatment on apple rootstocks under NaCl stress in terms of ^15^N accumulation **(A)** and ^15^N distribution ratio **(B)**. The error bars represent the SD, and the different letters indicate significant differences (*P*< 0.05).

Even after 15 days of treatment, the control treatment still showed the highest ^15^N accumulation in the entire seedlings. Comparatively, under SS conditions, the total ^15^N accumulation in the EBR-treated plants was 56.54% higher than in the non-EBR-treated plants (**Figure 6A**). We further analyzed the ^15^N distribution ratio in the seedlings under various treatments. As depicted in **Figure 6B**, the ^15^N distribution ratio of leaves under SS treatment was obviously lower than that in the control. The opposite trend, however, was observed in the roots. The leaves’ ^15^N distribution rate under the SS+EBR treatment reached 42.63%, which was 1.17 times that under the SS treatment.

### 3.7 Roots’ net NO_3_^−^ ion flow rate and relative expression of NRT genes

**Figure 7:**
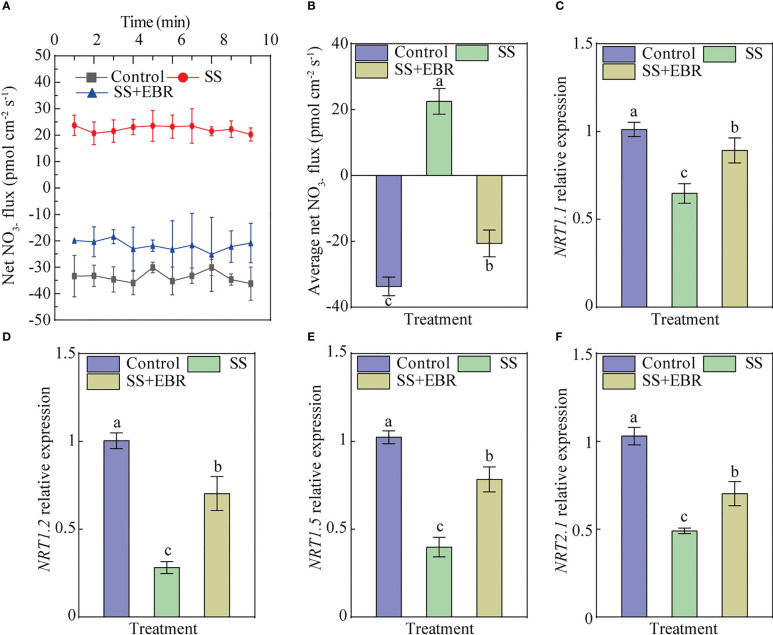
Net ${\text{NO}}_{3}^{-}$ fluxes in the roots of the seedlings for a 10-min period **(A)**, mean rate of ${\text{NO}}_{3}^{-}$ fluxes during the entire 10-min period **(B)** and the expression of the *NRT1.1* **(C)**, *NRT1.2* **(D)**, *NRT1.5* **(E)** and *NRT2.1* **(F)** genes in the roots of seedlings. The error bars represent the SD, and the different letters indicate significant differences (*P*< 0.05).

We measured the roots’ net NO_3_^−^ fluxes for a 10-min period and then averaged the values (**Figures 7A, B**). The findings demonstrated that the net fluxes of ${\text{NO}}_{3}^{-}$ of SS treatment exhibited a tendency to be excreted. Contrarily, the average ${\text{NO}}_{3}^{-}$ flux rates under SS+EBR presented a tendency to be absorbed, despite the average ${\text{NO}}_{3}^{-}$ influx rate being 0.61 times that of the control (**Figure 7B**).

The expression of *NRT1.1*, *NRT1.2*, *NRT1.5*, and *NRT2.1* treated by salt stress and EBR spraying treatment were analyzed. The finding demonstrate the SS-influenced decline in the four NRT genes expression. Notably, after 15 days of exogenous EBR administration, the decline in the expression of these genes was noticeably smaller than that under the SS treatment (**Figures 7C–F**).

## 4 Discussion

### 4.1 Changes in growth parameters and photosynthesis of M9T337 seedlings under different treatments

The negative effects induced by salt stress on seedling growth could be reflected in the dry weight. We observed that the dry weight of seedlings’ organs both decreased noticeably under SS conditions (**Figure 1E**), which was in line with the findings of Su et al. (2020) and Zheng et al. (2021). Exogenous EBR treatment increased the dry weight of the seedlings under NaCl stress, indicating that it could alleviate seedling growth inhibition induced by NaCl toxicity. The reason might be closely related with the improvement of the seedlings’ salt stress tolerance (**Figure 2**).

N is essential for the growth and fundamental metabolic processes in apples (Ge et al., 2018; Xing et al., 2021). It has been extensively documented that exogenous EBR treatment is crucial in influencing N absorption and metabolism activities in plants, especially in the stressful conditions (Wani et al., 2017; Xia et al., 2022; Xing et al., 2022). As the primary organs in plants for absorbing nutrients, roots with proper root morphology and high root activity are essential for N absorption (Liang B et al., 2018). Our results indicated that seedlings employed with exogenous EBR (0.2 mg/L) under SS condition exhibited higher root dry weight, root length, and root surface area as well as enhanced root activity than seedlings solely under SS treatment (**Figures 1B**–**D**), which was in accordance with the trend of N (^15^N) accumulation (**Figure 6B**). Leaf photosynthesis and leaves-to-roots translocation of photosynthates are fundamental for root growth and nutrient uptake (Hu et al., 2017; Li et al., 2018; Ren et al., 2020). The variations in root growth under various treatments may be due to the variations in leaf photosynthesis, photosynthesis-mediated C fixation and the restricted movement of photosynthates (**Figures 3**, **5**).

As an important parameter reflecting the intensity of photosynthesis, the chlorophyll concentration could be affected by salt stress in a number of ways, such as obstructing the chlorophyll biosynthesis pathway (Hoertensteiner, 2013), decreasing the amount of chlorophyll biosynthesis precursors (Yuan et al., 2018), and downregulating the chlorophyll biosynthesis-related genes expression (Turan and Tripathy, 2015). Consistent with the earlier findings, we discovered that the amount of chlorophyll decreased under SS treatment, indicating that the SS treatment inhibited leaf photosynthesis. However, when exogenous EBR was applied along with SS treatment, the chlorophyll content increased (**Figure 3A**). This might be because EBR has considerable potential for preventing the degradation of chlorophyll induced by high salt damage (Dong et al., 2017). Another reason might be the enhancement of the chlorophyll biosynthesis *via* BR-signalling transcription and translation (Honnerová et al., 2010). Moreover, exogenous EBR treatment weakened the salt stress toxicity on the *P*_n_ (**Figure 3B**). This agrees with the outcomes reported by Chen et al. (2015). The underlying reason may be connected with the alleviation of leaves’ ionic injury (high Na^+^: K^+^) induced by exogenous EBR treatment (**Figure 2H**). Moreover, after 15 days of exposure to SS, the *G*_s_ of M9T337 seedlings also drastically dropped, whereas the treatment with EBR elevated the value of *G*_s_ (**Figure 3C**), which was congruent with those obtained by Mafakheri et al. (2010) observed in chickpea. A higher *G*_s_ indicates that photosynthetic production increased as a consequence of an increased capacity of plants to take up CO_2_ (Lawlor and Cornic, 2002; Singh et al., 2013), which might explain why EBR-treated seedlings displayed a higher ^13^C assimilation rate and accumulation than non-EBR-treated plants under salt stress (**Figure 5**). We also observed the values of *F*_v_/*F*_m_, *Φ*PSII and *ETR* were decreased and the value of *NPQ* was increased in the SS treatment, compared with control (**Figures 3D**–**G**). This showed that salt stress decreased the light energy utilization and electron transport rate in seedlings but increased the heat dissipation of light energy. Exogenous EBR treatment in a salt stress environment, however, reduced these inhibitory effects (**Figures 3D**–**G**). Earlier studies have proven that the ionic injury induced by the over accumulation of Na^+^ in the salt-stressed plants was closely related with the formation of ROS (Tanveer et al., 2018). Moreover, the elevation of thermal dissipation under salt stress could not only decrease the photosynthetic efficiency (Fariduddin et al., 2014), but also enhance photooxidative damage by increasing the accumulation of reactive oxygen species (ROS) (Miller et al., 2010; Tofighi et al., 2017). Our findings showed that the administration of exogenous EBR under salt stress could obviously lower the contents of ${\text{O}}_{2}^{\cdot -}$ , H_2_O_2_ and MDA in leaves (**Figures 2A**–**C**) and elevate leaves’ SOD and CAT activities than non EBR-treated (**Figures 2E**–**G**), which was consistent with the outcomes obtained by Fariduddin et al. (2013) and Su et al. (2020). Therefore, the considerable potential of EBR to minimize ROS production could be another reason for the improvement in photosynthetic capacity of seedlings.

The intensity of the process of photosynthesis-mediated C fixation is closely connected with Rubisco activity (Scheibe et al., 1986; Wen et al., 2019; Lan et al., 2020). Our results showed that Rubisco activity in leaves of seedlings in a salt stress environment decreased considerably, which was consistent with the results obtained by Li et al. (2017). However, the reduction in Rubisco activity induced by NaCl stress was mitigated by exogenous EBR treatment (**Figure 4D**). These findings might offer a promising way to further explain why, under SS+EBR-treated seedlings displayed higher ^13^C accumulation as compared to seedlings treated solely with SS (**Figure 5A**). The analysis of the ^13^C distribution rate in the roots indicated that applying exogenous EBR under SS conditions enhanced the translocation of photosynthates from leaves to roots (**Figure 5B**). Previous studies have proved that exogenous EBR treatment under salt stress could alleviate seedlings’ ionic injury caused by high Na^+^: K^+^ *via* decreasing the content of Na^+^ and increasing the content of K^+^ (Su et al., 2020). The elevation of K^+^ content could be favor to the translocation of photosynthates from leaves to roots (Xu et al., 2020). Therefore, the translocation of photosynthates from leaves to roots might be closely related with the enhancement of K^+^ uptake caused by exogenous EBR treatment under salt stress. The changes of the translocation of photosynthates from leaves to roots could also explain the higher root dry weight under SS+EBR treatment as compared to only SS treatment, indicating the beneficial role of EBR in root growth against in a salt stress environment. This study demonstrated that the administration of exogenous EBR under SS condition influenced the assimilation and distribution of C by improving leaves’ salt stress tolerance and then strengthening photosynthesis and boosting the activity of enzymes involved in C assimilation. Increased photosynthetic product distribution to the root system encouraged root development and then improved N absorption.

### 4.2 Changes in N absorption, assimilation, and distribution of M9T337 under different treatments

Xing et al. (2021) noted that the roots’ N uptake could be directly exhibited *via* the positive or negative values of ${\text{NO}}_{3}^{-}$ flow rate. Studies in cucumber (Anwar et al., 2019) and Maize (Xing et al., 2022) showed that the application of EBR has a favorable influence on the roots’ N absorption in the stress environments through elevating the ${\text{NO}}_{3}^{-}$ influx rate. In line with the earlier studies, our results showed that the SS+EBR treatment caused a ${\text{NO}}_{3}^{-}$ influx into the roots, while the SS treatment caused a net ${\text{NO}}_{3}^{-}$ efflux from the roots (**Figures 7A, B**). This suggested that EBR could significantly enhance the N influx of seedlings under salt-stressed condition. Moreover, Rashid et al. (2018) and Chen et al. (2021) noted that the enhancement of root’s ability to absorb ${\text{NO}}_{3}^{-}$ was also closely related with the upregulation of the *NRT* genes. Therefore, the differences in *NRT* genes expression could be another reason for the changes in the N absorption under different treatments. In the current study, treatment with SS+EBR considerably elevated roots’ *MdNRT1.1*, *MdNRT1.2*, *MdNRT1.5*, and *MdNRT2.1* expression (**Figures 7C**–**F**), and then promoted ${\text{NO}}_{3}^{-}$ uptake.

Elevating the N assimilation-related enzymes activities is essential for enhancing the N assimilation of plants, and then improving plants’ NUE (Bajguz, 2000; Balkos et al., 2010; Coskun et al., 2017; Teng et al., 2017; Hou et al., 2019; Xia et al., 2022). Existing studies have stated that the plants’ N assimilation could be easily affected in the salt stressed environment, and exogenous EBR obviously alleviated the inhibition of N assimilation induced by salt stress through improving N assimilation enzymes activities, such as NR, GS, and GOGAT (Gupta et al., 2017; Wani et al., 2017). In our study, we discovered that SS treatment significantly reduced the value of NR, GS, and GOGAT activities, while SS+EBR treatment increased the activity of these enzymes (**Figures 4A**–**C**), suggesting that the exogenous EBR applications’ was propitious to enhancing the assimilation of N of seedling exposed in the salt-stressed condition.

The uptake and assimilation of nutrient, such as N, depends largely on the energy and C skeletons produced by photosynthesis (Fredes et al., 2019). Therefore, the strengthening in the uptake and assimilation of N by seedlings could also be related to the increment in photosynthesis and photosynthesis-mediated C fixation caused by exogenous EBR application in a salt-stressed condition. Moreover, Xu et al. (2020) noted that the higher NUE of seedlings was strongly associated with the increase in leaf N distribution rate. Increasing the distribution of ${\text{NO}}_{3}^{-}$ in the plants’ leaves could maximize the utilization of light energy for photosynthesis and its-mediated C fixation, thus enhancing the plants’ N assimilation (Reguera et al., 2013; Ren et al., 2021). The outcomes of the ^15^N labeling experiment indicated that under SS treatment, the ^15^N distribution ratio in the roots increased, and the lowest ^15^N distribution ratio was observed in the leaves. However, the leaves’ ^15^N distribution ratio was obviously increased compared with SS treatment, when exogenous EBR was applied under SS treatment (**Figure 6B**). This suggested that salt stress could inhibit roots-to-leaves translocation of N, while exogenous EBR application could alleviate the reduction in roots-to-leaves translocation. The reason may be associated with the change of *MdNRT1.5* between SS and SS+EBR treatments due to its role in regulating the roots-to-leaves translocation of seedlings’ ${\text{NO}}_{3}^{-}$ (Han et al., 2016; Xu et al., 2022).

## 5 Conclusion

**Figure 8:**
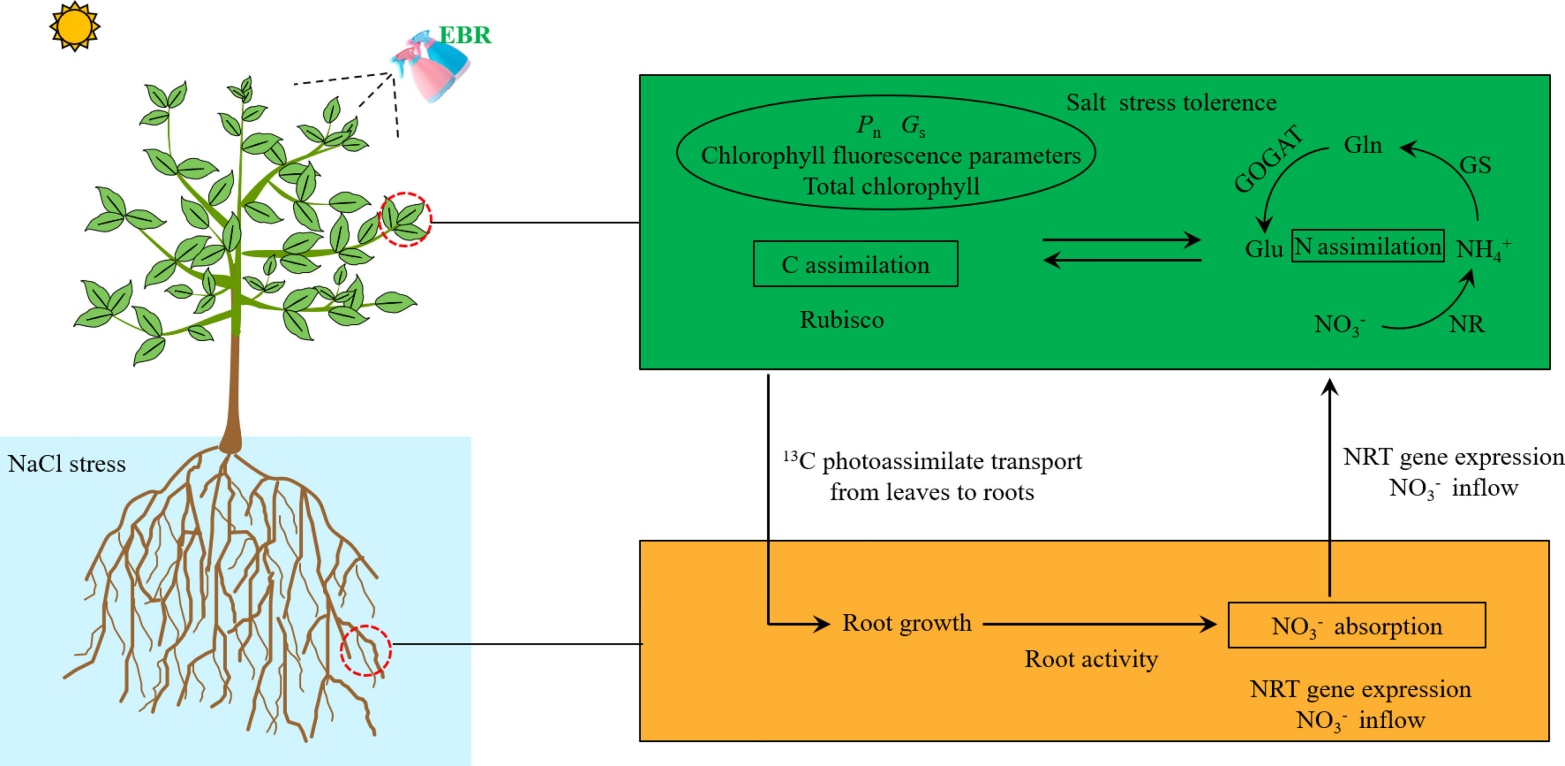
Schematic model displaying the role of EBR on nitrate nitrogen absorption and assimilation efficiency in salt-stressed apple seedling.

Our study revealed that apple dwarf rootstock (M9T337) seedlings treated with exogenous EBR under NaCl stress displayed the following characteristics (**Figure 8**): (i) more ideal root morphology and higher root activity; (ii) strengthened leaves’ salt stress tolerance, photosynthetic capacity and leaves to roots translocation of ^13^C; (iii) amplified root ${\text{NO}}_{3}^{-}$ ion inflow rate and improved nitrate transport; (iv) comparatively higher N metabolism-related enzyme activity; (v) enriched ^15^N translocation from the roots to the leaves; and (vi) increased ^15^N uptake. Overall, this study offers fresh perspectives into EBR-induced N absorption and assimilation in salt-stressed apple plants, with possible consequences for apple production.

## Data availability statement

The original contributions presented in the study are included in the article/**Supplementary Material**. Further inquiries can be directed to the corresponding author.

## Author contributions

Experiment design, BY, WG, and PN; methodology, BY, LW, and XX; experiment performance and data analysis, BY; manuscript preparation and writing, BY; writing and editing, BY, QG, and PN; funding acquisition, PN. All authors contributed to the article and approved the submitted version.

## References

[B1] Abbas S. Latif H. H. Elsherbiny E. A. (2013). Effect of 24-epibrassinolide on the physiological and genetic changes on two varieties of pepper under salt stress conditions. Pakistan J. Bot. 45, 1273–1284.

[B2] Abdelgadir E. M. Oka M. Fujiyama H. (2005). Nitrogen nutrition of rice plants under salinity. Biol. Plantarum. 49, 99–104. doi: 10.1007/s10535-005-0104-8

[B3] Ahammed G. J. Li X. Liu A. Chen S. (2020). Brassinosteroids in plant tolerance to abiotic stress. J. Plant Growth Regul. 39, 1–14. doi: 10.1007/s00344-020-10098-0

[B5] Anwar Y. Li C. Yu X. (2019). 24-epibrassinolide promotes ${\text{NO}}_{3}^{-}$ and ${\text{NH}}_{4}^{+}$ ion flux rate and *NRT1* gene expression in cucumber under suboptimal root zone temperature. BMC Plant Biol. 19, 225. doi: 10.1186/s12870-019-1838-3 31146677 PMC6543628

[B6] Bai L. Q. Deng H. H. Zhang X. C. Yu X. C. Li Y. S. (2016). Gibberellin is involved in inhibition of cucumber growth and nitrogen uptake at suboptimal root-zone temperatures. PloS One 11, e0156188. doi: 10.1371/journal.pone.0156188 27213554 PMC4877016

[B7] Bajguz A. (2000). Effect of brassinosteroids on nucleic acid and protein content in cultured cells of chlorella vulgaris. Plant Physiol. Biochem. 38, 209–215. doi: 10.1016/S0981-9428(00)00733-6

[B8] Balkos K. D. Britto D. T. Kronzucker H. J. (2010). Optimization of ammonium acquisition and metabolism by potassium in rice (*Oryza sativa* l. cv. IR-72). Plant Cell Environ. 33, 23–34. doi: 10.1111/j.1365-3040.2009.02046.x 19781010

[B9] Betzen B. M. Smart C. M. Maricle K. L. MariCle B. R. (2019). Effects of increasing salinity on photosynthesis and plant water potential in Kansas salt marsh species. Trans. Kans. Acad. Sci. 122, 49. doi: 10.1660/062.122.0105

[B12] Chen Q. Ding N. Peng L. Ge S. F. Jiang Y. M. (2017). Effects of different nitrogen application rates on ^15^ N-urea absorption, utilization, loss and fruit yield and quality of dwarf apple. Chin. J. Appl. Ecol. 28, 2247–2253. doi: 10.13287/j.1001-9332.201707.001 29741056

[B13] Chen T. W. Kahlen K. Stützel H. (2015). Disentangling the contributions of osmotic and ionic effects of salinity on stomatal, mesophyll, biochemical and light limitations to photosynthesis. Plant Cell Environ. 38 (8), 1528–1542. doi: 10.1111/pce.12504 25544985

[B10] Chen G. D. Wang L. Fabrice M. R. Tian Y. N. Qi K. J. Chen Q.. (2018). Physiological and nutritional responses of pear seedlings to nitrate concentrations. Front. Plant Sci. 9, 1679. doi: 10.3389/fpls.2018.01679 30515181 PMC6255940

[B11] Chen H. F. Zhang Q. Wang X. R. Zhang J. H. Ismail A. M. Zhang Z. H. (2021). Nitrogen form-mediated ethylene signal regulates root-to-shoot K^+^ translocation *via NRT1.5* . Plant Cell Environ. 44, 3576–3588. doi: 10.1111/pce.14182 34505300

[B14] Coskun D. Britto D. T. Kronzucker H. J. (2017). The nitrogen–potassium intersection: membranes, metabolism, and mechanism. Plant Cell Environ. 10, 2029–2041. doi: 10.1111/pce.12671 26524711

[B15] Dong Y. Wang W. Hu G. Chen W. Zhuge Y. Wang Z.. (2017). Role of exogenous 24-epibrassinolide in enhancing the salt tolerance of wheat seedlings. J. Soil Sci. Plant Nutr. 17, 554–569. doi: 10.4067/S0718-95162017000300001

[B16] Duran M. I. Gonzalez C. Acosta A. Olea A. F. Díaz K. Espinoza L. (2017). Synthesis of five known brassinosteroid analogs from hyodeoxycholic acid and their activities as plant-growth regulators. Int. J. Mol. Sci. 18, 516. doi: 10.3390/ijms18030516 28282853 PMC5372532

[B17] Erdal S. (2019). Melatonin promotes plant growth by maintaining integration and coordination between carbon and nitrogen metabolisms. Plant Cell Rep. 38, 1001–1012. doi: 10.1007/s00299-019-02423-z 31069499

[B18] Fariduddin Q. Khalil R. R. Mir B. A. Yusuf M. Ahmad A. (2013). 24-epibrassinolide regulates photosynthesis, antioxidant enzyme activities and proline content of *Cucumis sativus* under salt and/or copper stress. Environ. Monit. Assess. 185, 7845–7856. doi: 10.1007/s10661-013-3139-x 23443638

[B19] Fariduddin Q. Mir B. Yusuf M. Ahmad A. (2014). 24-epibrassinolide and/or putrescine trigger physiological and biochemical responses for the salt stress mitigation in *Cucumis sativus* l. Photosynthetica 52, 464–474. doi: 10.1007/s11099-014-0052-7

[B20] Fredes I. Moreno S. Díaz F. P. Guti´errez R. A. (2019). Nitrate signaling and the control of *Arabidopsis* growth and development. Curr. Opin. Plant Biol. 47, 112–118. doi: 10.1016/j.pbi.2018.10.004 30496968

[B21] Ge S. F. Zhu Z. L. Peng L. Chen Q. Jiang Y. M. (2018). Soil nutrient status and leaf nutrient diagnosis in the main apple producing regions in China. Hortic. Plant J. 4, 89–93. doi: 10.1016/j.hpj.2018.03.009

[B22] Gupta P. Srivastava S. Seth C. S. (2017). 24-epibrassinolide and sodium nitroprusside alleviate the salinity stress in *Brassica juncea* l. cv. *Varuna* through cross talk among proline, nitrogen metabolism and abscisic acid. Plant Soil. 411, 483–498. doi: 10.1007/s11104-016-3043-6

[B23] Han Y. L. Song H. X. Liao Q. Yu Y. Jian S. F. Lepo J. E.. (2016). Nitrogen use efficiency is mediated by vacuolar nitrate sequestration capacity in roots of *Brassica napus* . Plant Physiol. 170, 1684–1698. doi: 10.1104/pp.15.01377 26757990 PMC4775117

[B70] He J. L. Zhou J. T. Wan H. X. Zhuang X. L. Li H. F. Qin S. J.. (2020). Rootstocks-scion interaction affects cadmium accumulation and tolerance of *M*alus. Front. Plant Sci. 11, 1264. doi: 10.3389/fpls.2020.01264 32922429 PMC7457089

[B24] Hoagland D. R. Arnon D. I. (1950). The water-culture method for growing plants without soil. Calif. Agric. Exp. Station Circ. 347, 1–32. doi: 10.1016/S0140-6736(00)73482-9

[B25] Hoertensteiner S. (2013). Update on the biochemistry of chlorophyll breakdown. Plant Mol. Biol. 82 (6), 505–517. doi: 10.1007/s11103-012-9940-z 22790503

[B26] Honnerová J. Rothová O. Holá D. Kočová M. Kohout L. Kvasnica M. (2010). The exogenous application of brassinosteroids to zea mays (L.) stressed by long-term chilling does not affect the activities of photosystem 1 or 2. J. Plant Growth Regul. 29, 500–505. doi: 10.1007/s00344-010-9153-0

[B27] Hou W. F. Xue X. X. Li X. K. Khan M. R. Yan J. Y. Ren T.. (2019). Interactive effects of nitrogen and potassium on: grain yield, nitrogen uptake and nitrogen use efficiency of rice in low potassium fertility soil in China. Field Crop Res. 236, 14–23. doi: 10.1016/j.fcr.2019.03.006

[B28] Hu W. Coomer T. D. Loka D. A. Oosterhuis D. M. Zhou Z. (2017). Potassium deficiency affects the carbon-nitrogen balance in cotton leaves. Plant Physiol. Biochem. 115, 408–417. doi: 10.1016/j.plaphy.2017.04.005 28441628

[B29] Hu W. Jiang N. Yang J. Meng Y. Wang Y. Chen B.. (2016a). Potassium (K) supply affects K accumulation and photosynthetic physiology in two cotton (*Gossypium hirsutum* l.) cultivars with different K sensitivities. Field Crop Res. 196, 51–63. doi: 10.1016/j.fcr.2016.06.005

[B30] Hu W. Zhao W. Yang J. Oosterhuis D. M. Loka D. A. Zhou Z. (2016b). Relationship between potassium fertilization and nitrogen metabolism in the leaf subtending the cotton (*Gossypium hirsutum* l.) boll during the boll development stage. Plant Physiol. Biochem. 101, 113–123. doi: 10.1016/j.plaphy.2016.01.019 26874296

[B31] Hussain S. Iqbal N. Brestic M. Raza M. A. Pang T. Langham D. R.. (2019). Changes in morphology, chlorophyll fluorescence performance and rubisco activity of soybean in response to foliar application of ionic titanium under normal light and shade environment. Sci. Total Environ. 658, 626–637. doi: 10.1016/j.scitotenv.2018.12.182 30580217

[B32] Jia X. Wang H. Svetla S. Zhu Y. Hu Y. Cheng L.. (2019). Comparative physiological responses and adaptive strategies of apple *Malus halliana* to salt, alkali and saline-alkali stress. Sci. Hortic. 245, 154–162. doi: 10.1016/j.scienta.2018.10.017

[B33] Jia X. Zhu Y. Zhang R. Zhu Z. Zhao T. Cheng L.. (2020). Ionomic and metabolomic analyses reveal the resistance response mechanism to saline-alkali stress in *Malus halliana* seedlings. Plant Physiol. Biochem. 147, 77–90. doi: 10.1016/j.plaphy.2019.12.001 31846851

[B34] Kanwar M. K. Bajguz A. Zhou J. Bhardwaj R. (2017). Analysis of brassinosteroids in plants. J. Plant Growth Regul. 36, 1002–1030. doi: 10.1007/s00344-017-9732-4

[B35] Karlidag H. Yildirim E. Turan M. (2011). Role of 24-epibrassinolide in mitigating the adverse effects of salt stress on stomatal conductance, membrane permeability, and leaf water content, ionic composition in salt stressed strawberry (*Fragaria*×*ananassa*). Sci. Hortic. (Amst.) 130, 133–140. doi: 10.1016/j.scienta.2011.06.025

[B36] Kawanabe S. Zhu T. C. (1991). Degeneration and conservation of aneurolepidium chinese grassland in northern China. J. Jpn. Grassland Sci. 37, 91–99. doi: 10.14941/GRASS.37.91

[B37] Khan A. L. Hamayun M. Ahmad N. Hussain J. Kang S. M. Kim Y. H.. (2011). Salinity stress resistance offered by endophytic fungal interaction between penicillium minioluteum lhl09 and glycine max. L. J. Microbiol. Biotechnol. 21, 893–902. doi: 10.4014/jmb.1103.03012 21952365

[B38] Krishna P. (2003). Brassinosteroid-mediated stress responses. J. Plant Growth Regul. 22, 289–297. doi: 10.1007/s00344-003-0058-z 14676968

[B39] Krishnamurthy S. Gautam R. Sharma P. Sharma D. (2016). Effect of different salt stresses on agro-morphological traits and utilisation of salt stress indices for reproductive stage salt tolerance in rice. Field Crop Res. 190, 26–33. doi: 10.1016/j.fcr.2016.02.018

[B40] Lan G. Jian C. Wang G. Sun Y. Sun Y. (2020). Effects of dopamine on growth, carbon metabolism, and nitrogen metabolism in cucumber under nitrate stress. Sci. Hortic. 260, 108790. doi: 10.1016/j.scienta.2019.108790

[B41] Lawlor D. W. Cornic G. (2002). Photosynthetic carbon assimilation and associated metabolism in relation to water deficits in higherplants. Plant Cell Environ. 25, 275–294. doi: 10.1046/j.0016-8025.2001.00814.x 11841670

[B42] Li H. Chang J. Chen H. Wang Z. Gu X. Wei C.. (2017). Exogenous melatonin confers salt stress tolerance to watermelon by improving photosynthesis and redox homeostasis. Front. Plant Sci. 8, 295. doi: 10.3389/fpls.2017.00295 28298921 PMC5331065

[B43] Li S. Tian Y. Wu K. Ye Y. Yu J. Zhang J.. (2018). Modulating plant growth–metabolism coordination for sustainable agriculture. Nature 560, 595–600. doi: 10.1038/s41586-018-0415-5 30111841 PMC6155485

[B45] Liang W. Ma X. Wan P. Liu L. (2018). Plant salt-tolerance mechanism: A review. Biochem. Bioph. Res. Co. 495, 286–291. doi: 10.1016/j.bbrc.2017.11.043 29128358

[B44] Liang B. Ma C. Zhang Z. Wei Z. Gao T. Zhao Q.. (2018). Long-term exogenous application of melatonin improves nutrient uptake fluxes in apple plants under moderate drought stress. Environ. Exp. Bot. 155, 650–661. doi: 10.1016/j.envexpbot.2018.08.016

[B46] Mafakheri A. Siosemardeh A. Bahramnejad B. Struik P. C. Sohrabi Y. (2010). Effect of drought stress on yield, proline and chlorophyll contents in three chickpea cultivars. Aust. J. Crop Sci. 4, 580–585. doi: 10.1007/s12230-010-9149-0

[B47] Miller G. Suzuki N. Ciftci-Yilmaz S. Mittler R. (2010). Reactive oxygen species homeostasis and signalling during drought and salinity stresses. Plant Cell Environ. 33, 453–467. doi: 10.1111/j.1365-3040.2009.02041.x 19712065

[B48] Mir B. A. Khan T. A. Fariduddin Q. (2015). 24-epibrassinolide and spermidine modulate photosynthesis and antioxidant systems in *Vigna radiata* under salt and zinc stress. Int. J. 3, 592–608.

[B49] Nazar R. Iqbal N. Masood A. Syeed S. Khan N. A. (2011). Understanding the significance of sulfur in improving salinity tolerance in plants. Environ. Exp. Bot. 70, 80–87. doi: 10.1016/j.envexpbot.2010.09.011

[B50] Peres A. Soares J. S. Tavares R. G. Righetto G. Zullo M. A. T. Mandava N. B.. (2019). Brassinosteroids, the sixth class of phytohormones: a molecular view from the discovery to hormonal interactions in plant development and stress adaptation. Int. J. Mol. Sci. 20 (2), 331. doi: 10.3390/ijms20020331 30650539 PMC6359644

[B51] Rashid M. Bera S. Medvinsky A. B. Sun G. Q. Li B. L. Chakraborty A. (2018). Adaptive regulation of nitrate transceptor NRT1.1 in fluctuating soil nitrate conditions. iScience 2, 41–50. doi: 10.1016/j.isci.2018.03.007 30428377 PMC6135930

[B52] Reguera M. Peleg Z. Abdel-Tawab Y. M. Tumimbang E. B. Delatorre C. A. Blumwald E. (2013). Stress-induced cytokinin synthesis increases drought tolerance through the coordinated regulation of carbon and nitrogen assimilation in rice. Plant Physiol. 163, 1609–1622. doi: 10.1104/pp.113.227702 24101772 PMC3850209

[B53] Ren J. Xie T. Wang Y. Li H. Liu T. Zhang S.. (2020). Coordinated regulation of carbon and nitrogen assimilation confers drought tolerance in maize (*Zea mays* l.). Environ. Exp. Bot. 176, 104086. doi: 10.1016/j.envexpbot.2020.104086

[B54] Ren J. Yang X. Ma C. Wang Y. Zhao J. (2021). Melatonin enhances drought stress tolerance in maize through coordinated regulation of carbon and nitrogen assimilation. Plant Physiol. Biochem. 167, 958–969. doi: 10.1016/j.plaphy.2021.09.007 34571389

[B55] Sairam R. (1994). Effects of homobrassinolide application on plant metabolism and grain yield under irrigated and moisture-stress conditions of two wheat varieties. J. Plant Growth Regul. 14, 173–181. doi: 10.1007/BF00025220

[B56] Scheibe R. Fickenscher K. Ashton A. R. (1986). Studies on the mechanism of the reductive activation of NADP-malate dehydrogenase by thioredoxin m and low molecular weight thiols. Biochim. Biophys. Acta 870, 191–197. doi: 10.1016/0167-4838(86)90221-9

[B57] Sha J. C. Jia Z. H. Xu X. X. Hou X. Li B. Y. Ge S. F.. (2019). Effects of nitrogen application levels on translocation and distribution of ^13^Cphotosynthate and ^15^N to fruit from leaves of apple tree. J. Appl. Ecol. 30, 1373–1379. doi: 10.13287/j.1001-9332.201904.011 30994301

[B58] Shabala S. (2009). Salinity and programmed cell death: unravelling mechanisms for ion specific signalling. J. Exp. Bot. 60, 709–712. doi: 10.1093/jxb/erp013 19269993

[B59] Shahbaz M. Ashraf M. (2008). Does exogenous application of 24-epibrassinolide ameliorate salt induced growth inhibition in wheat (*Triticum aestivum* l.)? J. Plant Growth Regul. 55, 51–64. doi: 10.1007/s10725-008-9262-y

[B60] Shahid M. A. Balal R. M. Pervez M. A. Garcia-Sanchez F. Gimeno V. Abbas T.. (2014). Treatment with 24-epibrassinolide mitigates NaCl-induced toxicity by enhancing carbohydrate metabolism, osmolyte accumulation, and antioxidant activity in *Pisum sativum.* Turk. J. Bot. 38, 511–525. doi: 10.3906/bot-1304-45

[B61] Shu S. Tang Y. Yuan Y. Sun J. Zhong M. Guo S. (2016). The role of 24-epibrassinolide in the regulation of photosynthetic characteristics and nitrogen metabolism of tomato seedlings under a combined low temperature and weak light stress. Plant Physiol. Biochem. 107, 344–353. doi: 10.1016/j.plaphy.2016.06.021 27362298

[B62] Singh S. K. Badgujar G. Reddy V. R. Fleisher D. H. Bunce J. A. (2013). Carbon dioxide diffusion across stomata and mesophyll and photo-biochemical processes as affected by growth CO_2_ and phosphorus nutrition in cotton. J. Plant Physiol. 170, 801–813. doi: 10.1016/j.jplph.2013.01.001 23384758

[B63] Soylemez S. Kaya C. Dikilitas S. K. (2017). Promotive effects of epibrassinolide on plant growth, fruit yield, antioxidant, and mineral nutrition of saline stressed tomato plants. Pakistan J. Bot. 49, 1655–1661.

[B64] Su Q. Zheng X. Tian Y. Wang C. (2020). Exogenous brassinolide alleviates salt stress in *Malus hupehensis* rehd. by regulating the transcription of NHX-type NA^+^ (K^+^)/H^+^ antiporters. Front. Plant Sci 11, 38. doi: 10.3389/fpls.2020.00038 32117377 PMC7016215

[B65] Talaat N. B. Shawky B. T. (2012). 24-epibrassinolide ameliorates the saline stress and improves the productivity of wheat (*Triticum aestivum* l.). Environ. Exp. Bot. 82, 80–88. doi: 10.1016/j.envexpbot.2012.03.009

[B66] Tanveer M. Shahzad B. Sharma A. Biju S. Bhardwaj R. (2018). 24-epibrassinolide; an active brassinolide and its role in salt stress tolerance in plants: a review. Plant Physiol. Biochem. 130, 69–75. doi: 10.1016/j.plaphy.2018.06.035 29966934

[B67] Teng Y. Cui H. Wang M. Liu X. (2017). Nitrate reductase is regulated by CIRCADIAN CLOCK-ASSOCIATED1 in *Arabidopsis thaliana* . Plant Soil. 416, 477–485. doi: 10.1007/s11104-017-3208-y

[B68] Tofighi C. Khavari-Nejad R. A. Najafi F. Razavi K. Rejali F. (2017). Responses of wheat plants to interactions of 24-epibrassinolide and glomus mosseae in saline condition. Physiol. Mol. Boil. Pla. 23, 557–564. doi: 10.1007/s12298-017-0439-6 PMC5567700 28878494

[B69] Turan S. Tripathy B. C. (2015). Salt-stress induced modulation of chlorophyll biosynthesis during de-etiolation of rice seedlings. Physiol. Plant 153 (3), 477–491. doi: 10.1111/ppl.12250 25132047

[B71] Wang F. Sha J. C. Chen Q. Xu X. X. Zhu Z. L. Ge S. F.. (2020a). Exogenous abscisic acid regulates distribution of ^13^C and ^15^N and anthocyanin synthesis in ‘Red fuji’ apple fruit under high nitrogen supply. Front. Plant Sci. 10, 1738. doi: 10.3389/fpls.2019.01738 32063908 PMC6997889

[B72] Wang F. Xu X. X. Jia Z. H. Hou X. Chen Q. Sha J. C.. (2020b). Nitrification inhibitor 3,4-dimethylpyrazole phosphate application during the later stage of apple fruit expansion regulates soil mineral nitrogen and tree carbon–nitrogen nutrition, and improves fruit quality. Front. Plant Sci. 11, 764. doi: 10.3389/fpls.2020.00764 32582269 PMC7285628

[B73] Wang N. Xu H. F. Jiang S. H. Zhang Z. Y. Lu N. L. Qiu H. R.. (2017). MYB12 and MYB22 play essential roles in proanthocyanidin and flavonol synthesis in red-fleshed apple (*Malus sieversii* f.*niedzwetzkyana*). Plant J. 90 (2), 276–292. doi: 10.1016/j.scienta.2017.06.063 28107780

[B74] Wani A. Tahir I. Ahmad S. Dar R. Nisar S. (2017). Efficacy of 24-epibrassinolide in improving the nitrogen metabolism and antioxidant system in chickpea cultivars under cadmium and/or NaCl stress. Sci. Hortic. 225, 48–55.

[B75] Wen B. B. Li C. Fu X. L. Li D. M. Gao D. S. (2019). Effects of nitrate deficiency on nitrate assimilation and chlorophyll synthesis of detached apple leaves. Plant Physiol. Biochem. 142, 363–371. doi: 10.1016/j.plaphy.2019.07.007 31398585

[B76] Wu X. X. Ding H. D. Zhu Z. W. Yang S. J. Zha D. S. (2012). Effects of 24-epibrassinolide on photosynthesis of eggplant (*Solanum melongena* l.) seedlings under salt stress. Afr. J. Biotechnol. 11, 8665–8671. doi: 10.5897/AJB11.3416

[B77] Xia H. Liu X. Wang Y. Lin Z. Deng H. Wang J.. (2022). 24-epibrassinolide and nitric oxide combined to improve the drought tolerance in kiwifruit seedlings by proline pathway and nitrogen metabolism. Sci. Hortic. 297, 110929. doi: 10.1016/j.scienta.2022.110929

[B78] Xing J. Wang Y. Yao Q. Zhang Y. Zhang M. Li Z. (2022). Brassinosteroids modulate nitrogen physiological response and promote nitrogen uptake in maize (*Zea mays* l.). Crop J. 10, 166–176. doi: 10.1016/j.cj.2021.04.004

[B79] Xing Y. Zhu Z. Wang F. Zhang X. Li B. Liu Z.. (2021). Role of calcium as a possible regulator of growth and nitrate nitrogen metabolism in apple dwarf rootstock seedlings. Sci. Hortic. 276 (2021), 109740. doi: 10.1016/j.scienta.2020.109740

[B80] Xu X. X. Du X. Wang F. Sha J. C. Chen Q. Tian G.. (2020). Effects of potassium levels on plant growth, accumulation and distribution of carbon, and nitrate metabolism in apple dwarf rootstock seedlings. Front. Plant Sci. 11, 904. doi: 10.3389/fpls.2020.00904 32655607 PMC7325393

[B81] Xu X. X. Wang F. Xing Y. Liu J. Q. Lv M. X. Meng H.. (2022). Appropriate and constant potassium supply promotes the growth of M9T337 apple rootstocks by regulating endogenous hormones and carbon and nitrogen metabolism. Front. Plant Sci. 13, 827478. doi: 10.3389/fpls.2022.827478 35371125 PMC8967362

[B82] Yang J. Guo X. Li W. H. Chen P. H. Cheng Y. P. Ma F. W.. (2021). MdCCX2 of apple functions positively in modulation of salt tolerance. Environ. Exp. Bot. 192, 104663. doi: 10.1016/j.envexpbot.2021.104663

[B83] Yin R. Bai T. Ma F. Wang X. Li Y. Yue Z. (2010). Physiological responses and relative tolerance by Chinese apple rootstocks to NaCl stress. Sci. Hortic. 126, 247–252. doi: 10.1016/j.scienta.2010.07.027

[B84] Yuan R. N. Shu S. Guo S. R. Sun J. Wu J. Q. (2018). The positive roles of exogenous putrescine on chlorophyll metabolism and xanthophyll cycle in salt-stressed cucumber seedlings. Photosynthetica 56 (2), 557–566. doi: 10.1007/s11099-017-0712-5

[B85] Yusuf M. Fariduddin Q. Ahmad A. (2012). 24-epibrassinolide modulates growth, nodulation, antioxidant system, and osmolyte in tolerant and sensitive varieties of *Vigna radiata* under different levels of nickel: a shotgun approach. Plant Physiol. Biochem. 57, 143–153. doi: 10.1016/j.plaphy.2012.05.004 22705589

[B86] Zhao B. T. Zhu X. F. Jung J. H. Xuan Y. H. (2016). Effect of brassinosteroids on ammonium uptake *via* regulation of ammonium transporter and N metabolism genes in *Arabidopsis*, biol. Plant 60, 563–571. doi: 10.1016/j.scienta.2020.109740

[B87] Zheng X. D. Li Y. Q. Xi X. L. Ma C. Q. Sun Z. J. Yang X. Q.. (2021). Exogenous strigolactones alleviate KCl stress by regulating photosynthesis, ROS migration and ion transport in *Malus hupehensis* rehd. Plant Physiol. Biochem. 159, 113–122. doi: 10.1016/j.plaphy.2020.12.015 33359960

[B88] Zheng X. D. Xi X. L. Li Y. Q. Sun Z. J. Ma C. Q. Han M. S.. (2022). Effects and regulating mechanism of exogenous brassinosteroids on the growth of *Malus hupehensis* under saline-alkali stress. Acta Hortic. Sinica. 49 (7), 1404–1414. doi: 10.16420/j.issn.0513-353x.2021-0499

[B89] Zhu X. Q. Zhou P. Miao P. Wang H. Y. Bai X. L. Chen Z. J.. (2022). Nitrogen use and management in orchards and vegetable fields in china: challenges and solutions. Front. Agr. Sci. Eng. 9 (3), 386−395. 10.15302/J-FASE-2022443

