# Supplementary material for: Exogenous 24-epibrassinolide promoted growth and nitrogen absorption and assimilation efficiency of apple seedlings under salt stress
Source: Front Plant Sci. 2023 Apr 14;14:1178085. doi: 10.3389/fpls.2023.1178085 (PMC10140579; doi:10.3389/fpls.2023.1178085)
Supplement: Supplementary file 1 [file Table_1.docx]

**Table S1** Primers used for qRT-PCR.

| **Gene name** | **Primer-Forward** | **Primer-Reverse** |
| --- | --- | --- |
| *NRT1.1* ^a^ | 5’-CTGGCTGGTCCCACAGTTCTT-3’ | 5’- CTTCATTCCTTTCGGGCACTC-3’ |
| *NRT1.2* ^a^ | 5’-TTAATTGCTGCCACACTTCATAG-3’ | 5’- CACGATGTTTGGTTCTGATACTTC-3’  -3’ |
| *NRT1.5* ^a^ | 5’-AACAAGACAATGCGACAG-3’ | 5’-GATGACAGTGACAACGATAC-3’ |
| *NRT2.1* ^a^ | 5’-GCTGTACTCTTCCTGTGACTTT-3’ | 5’- CGTCGACTTCTCGACATCTTT-3’ |
| *β-actin* ^b^ | 5’-TACTCAGCTTTGGCAATCCACATC-3’ | 5’-TGACCGAATGAGCAAGGAAATTACT-3’ |

a: From Xu et al. Appropriate and Constant Potassium Supply Promotes the Growth of M9T337 Apple Rootstocks by Regulating Endogenous Hormones and Carbon and Nitrogen Metabolism. Frontiers in Plant Science. 2022, 13, 827478.

b: From Wang et al. MYB12 and MYB22 play essential roles in proanthocyanidin and flavonol synthesis in red-fleshed apple (*Malus sieversii f.niedzwetzkyana*). The Plant Journal, 2017, 90 (2): 276-292.
